# Establishment of Lipofection for Studying miRNA Function in Human Adipocytes

**DOI:** 10.1371/journal.pone.0098023

**Published:** 2014-05-21

**Authors:** Eveliina Enlund, Simon Fischer, René Handrick, Kerstin Otte, Klaus-Michael Debatin, Martin Wabitsch, Pamela Fischer-Posovszky

**Affiliations:** 1 Division of Pediatric Endocrinology and Diabetes, Department of Pediatrics and Adolescent Medicine, Ulm University Medical Center, Ulm, Germany; 2 Institute of Applied Biotechnology, University of Applied Sciences Biberach, Biberach, Germany; 3 Department of Pediatrics and Adolescent Medicine, Ulm University Medical Center, Ulm, Germany; Memorial Sloan Kettering Cancer Center, United States of America

## Abstract

miRNA dysregulation has recently been linked to human obesity and its related complications such as type 2 diabetes. In order to study miRNA function in human adipocytes, we aimed for the modulation of mature miRNA concentration in these cells. Adipocytes, however, tend to be resistant to transfection and there is often a need to resort to viral transduction or electroporation. Our objective therefore was to identify an efficient, non-viral transfection reagent capable of delivering small RNAs into these cells. To achieve this, we compared the efficiencies of three transfection agents, Lipofectamine 2000, ScreenFect A and BPEI 1.2 k in delivering fluorescent-labelled siRNA into human Simpson-Golabi-Behmel syndrome (SGBS) preadipocytes and adipocytes. Downregulation of a specific target gene in response to miRNA mimic overexpression was assayed in SGBS cells and also in *ex vivo* differentiated primary human adipocytes. Our results demonstrated that while all three transfection agents were able to internalize the oligos, only lipofection resulted in the efficient downregulation of a specific target gene both in SGBS cells and in primary human adipocytes. Lipofectamine 2000 outperformed ScreenFect A in preadipocytes, but in adipocytes the two reagents gave comparable results making ScreenFect A a notable new alternative for the gold standard Lipofectamine 2000.

## Introduction

The study of adipose tissue biology is becoming increasingly important as obesity and its related comorbidities, including type 2 diabetes, cardiovascular disease and certain cancers, are threatening the health of a growing number of people worldwide. The human Simpson-Golabi-Behmel syndrome (SGBS) preadipocyte cell strain [1], [2] is a unique, not immortalized cell model characterized by a high capacity for adipogenic differentiation. The cells display typical morphological, molecular, and functional characteristics of mature adipocytes *in vitro* and thus offer the opportunity to study various aspects of human adipocyte biology [1], [2]. Suppression of specific genes in order to identify components necessary for a particular cellular process or an event is a crucial tool in many studies. An elegant way to achieve this is RNA interference [3] where small, non-coding RNA species such as small interfering RNA (siRNA) and the genomically encoded microRNA (miRNA) modulate gene expression typically by causing the degradation of a complementary mRNA molecule. This approach, however, is often hindered in adipocytes because of inefficient transfection rates. Genetic modification in SGBS adipocytes is typically achieved via viral transduction [4], [5] or electroporation [6] but the disadvantages of these methods, for example the high reagent and equipment cost, growth arrest and possible cell damage associated with electroporation and the complexity and biosafety issues related with viruses, make them undesirable especially for high-throughput screens.

Since their initial discovery in the early 1990's, miRNAs have now been established as a well conserved and distinct class of gene expression regulators likely to be involved in most biological processes. In adipocytes, miRNAs have been shown to regulate adipogenic differentiation and lipid metabolism [7], [8], [9] in studies often conducted in the context of insulin resistance and obesity. Growing evidence is also suggesting that unique miRNA signatures detectable from plasma samples could exist for diseases like diabetes [10], [11]. A thorough understanding of the physiological function of these molecules is therefore of great interest as it will allow the development of novel miRNA based biomarkers and therapeutics.

We sought to establish a method to mimic the overexpression of miRNAs in human SGBS cells and also in human primary adipocytes. Lipid-based transfection would offer the simplest and most readily available means for RNA oligo delivery. Here we compare the efficiency of two cationic lipofection agents, the extensively used Lipofectamine 2000 and a more novel compound ScreenFect A, in delivering siRNA and functional miRNA into adherent human preadipocytes and adipocytes. Included in the comparison is also a cationic polymer branched 1.2 kDa polyethylenimine (BPEI 1.2 k) as it was demonstrated as an efficient small RNA agent in earlier studies [12], [13].

## Materials and Methods

### Materials

Cell culture reagents were purchased from Invitrogen (Darmstadt, Germany) and chemicals if not otherwise stated were from Sigma-Aldrich (München, Germany). Rosiglitazone was obtained from Cayman Europe (Tallinn, Estonia). Collagenase was from Sigma-Aldrich. Alexa Fluor 488 labelled, Alexa Fluor 647 labelled and the non-labelled control siRNAs as well as the miR-1 mimic were products of QIAGEN (Hilden, Germany). Lipofectamine 2000 was obtained from Invitrogen, ScreenFect A reagent from InCella (Eggenstein-Leopoldshafen, Germany) and the branched polyethylenimine from Polysciences Inc. (Eppelheim, Germany). Calcein-AM and DAPI were obtained from Invitrogen. The rabbit polyclonal twinfilin-1 primary antibody was from Cell Signaling Technology (Danvers, MA, USA) and the mouse monoclonal α-tubulin antibody from Calbiochem (Merck Millipore, Darmstadt, Germany). Horseradish peroxidase conjugated secondary antibodies were purchased from Santa Cruz Biotechnology (Heidelberg, Germany).

### Cell Culture

SGBS preadipocytes were cultured in DMEM/Ham's F12 (1∶1) containing 33 µM biotin, 17 µM pantothenate, 100 U/L penicillin, 0.1 mg/L streptomycin and 10% FBS. For differentiation, preadipocytes were seeded in a 12-well plate at a density of approximately 5300 cells/cm^2^ and grown to near confluency. Adipogenic differentiation was induced by washing the cells twice with PBS and culturing them in serum free medium (0 F) supplemented with 10 µg/ml iron-poor transferrin, 20 nM insulin, 0.2 nM triiodothyronine, and 100 nM cortisol. For the first four days 2 µM rosiglitazone, 250 µM IBMX, and 25 nM dexamethasone were added. The SGBS adipocytes were used in transfection experiments on day ten post-induction when the rate of adipogenic differentiation was >80%.

The rate of adipogenic differentiation was determined using a net micrometer by counting the number of preadipocytes and adipocytes and calculating the percentage of mature adipocytes. An adipocyte is defined by at least five clearly visibly lipid droplets.

Subcutaneous white adipose tissue samples were obtained from three female patients undergoing either abdominal or breast reduction surgeries (age 43±8 years, BMI 30±4 kg/m^2^). All experimental procedures were approved by the ethical committee of the University of Ulm, Germany, and performed in accordance with the Declaration of Helsinki guidelines. All patients gave their informed, written consent. Preadipocytes were prepared from the tissue samples by collagenase digestion according to a previously described protocol [14]. The cells were cultured and differentiated as SGBS cells (see above).

### Transfection

SGBS preadipocytes were seeded in a 12-well plate at a density of 7900 cells/cm^2^ and transfected the following day with final concentrations of 20 nM si/miRNA and 0.66 µl/cm^2^ Lipofectamine 2000, 0.53 µl/cm^2^ ScreenFect A and 0.13 µl/cm^2^ BPEI 1.2 k per each well. Prior to transfection, medium was replaced with fresh growth medium. For one well, the transfection agents and the mi/siRNA were first diluted separately in 50 µl of optiMEM (Lipofectamine 2000 and BPEI 1.2 k) or dilution buffer (ScreenFect A). Following a five minute incubation at room temperature, the diluted mi/siRNA was added to the transfection agent to yield a total volume of 100 µl. Complex formation was allowed to proceed at room temperature for 10 minutes (BPEI 1.2 k) or for 20 minutes (Lipofectamine 2000 and ScreenFect A). The complexes were added drop wise on the cells and the plates incubated at 37°C, 5% CO_2_. Transfection efficiency and oligonucleotide functionality were assessed 48 hours after transfection by detection of fluorescent labelled siRNA and qPCR and western blot analyses of miRNA target levels. SGBS adipocytes and primary human adipocytes were transfected as described for the preadipocytes on day eight post-induction of adipogenic differentiation.

### Detection of Fluorescently Labelled siRNA

#### Microscopy

SGBS preadipocytes were seeded on 4-chamber culture slides (BD Falcon, Heidelberg, Germany) and transfected the following day with Alexa Fluor 488 labelled, non-targeting siRNA (20 nM final concentration) as described above for the 12-well plate format. Here the transfection reagents and the siRNA were diluted in 25 µl of optiMEM/dilution buffer giving a total volume of 50 µl once combined. 48 hours after transfection, the cells were fixed and mounted with DAKO fluorescence mounting medium (DAKO, Hamburg, Germany) containing 1 µg/ml DAPI. Fluorescent detection of the labelled siRNA and the cell nuclei were carried out using a Keyence BIOREVO BZ-9000 fluorescence microscope and the BZII Viewer and Analyzer softwares. SGBS adipocytes were differentiated on the culture slides and transfected as described for the preadipocytes on day eight post-induction of adipogenic differentiation.

#### Flow Cytometry

At 48 hours post-transfection with an Alexa Fluor 488 labelled siRNA and a non-labelled control siRNA (20 nM final concentration), the cells were harvested and the percentage of positive cells was determined by flow cytometry in fluorescence channel F1 (FACSCalibur, Becton Dickinson, Heidelberg, Germany).

### Viability Assays

#### MTT Assay

Cells were treated with delivery agent alone or were transfected with a non-targeting control siRNA at a final concentration of 20 nM. Cell viability was determined 48 hours post-transfection after 3 h incubation at 37°C with the colorimetric substrate thiazolyl blue tetrazolium bromide (MTT, Sigma-Aldrich). Isopropanol was added, and the crystals were allowed to solubilise by incubation for 30 minutes at room temperature with constant shaking. Absorbance at λ = 550 nm was measured using a plate reader (EL800, Bio-tek Instruments GmbH, Bad Friedrichshall, Germany).

#### MACSQuant Analysis

At 48 hours post-transfection, cell viability was assessed using a Miltenyi Biotec MACSQuant Analyzer and the MACSquantify software. Cells were treated with delivery agent alone or were transfected using Alexa Fluor 647 labelled control siRNA at a final concentration of 20 nM. Cells were harvested and incubated with Calcein-AM (250 nM) in PBS at 37°C for 20 minutes to stain viable cells prior to analysis.

#### FSC/SSC analysis

With automated flow cytometry (FACScan, Becton Dickinson, Heidelberg, Germany), apoptotic cells were differentiated from viable cells based on their differing forward (FSC) and side (SSC) light scattering characteristics. Specific apoptosis was calculated as follows: 100 × (experimental DNA fragmentation (%)–spontaneous DNA fragmentation (%))/(100%–spontaneous DNA fragmentation (%)).

### Reverse transcription and quantitative PCR (qPCR)

To asses the effect of miR-1 over-expression on its target mRNA, cells were transfected either using a non-targeting siRNA or a miR-1 mimic at a final concentration of 20 nM. 48 hours after transfection, cell monolayers were rinsed with PBS. Total RNA was isolated using peqGOLD TriFast reagent and HP Total RNA Kit (PEQLAB Biotechnologie GmbH, Erlangen, Germany) according to manufacturer's protocol. Reverse transcription was performed using SuperScript II Reverse Transcriptase (Invitrogen) with random primers (Invitrogen) at 25°C for 15 min, 42°C for 50 min and 70°C for 15 min. LightCycler FastStart DNA MasterPLUS SYBR Green I kit and a carousel-based LightCycler 2.0 system from Roche were used to perform qPCR. Amplification was achieved with denaturation for 10 s at 95°C, annealing for 5 s at 60°C, and elongation for 15 s at 72°C for 40 cycles. All primers were from Thermo Fischer Scientific GmbH. Sequences for the twinfilin-1 forward and reverse primers were 5′-GGT GTG GAC ACT AAG CAT CAA ACA CTA CAA GG-3′ and 5′-ATC TAT TTC CAA CTG CAC ATA GTT GAG CTG TC-3′ respectively. PPARγ forward and reverse primers had the sequence 5′-GAT CCA GTG GTT GCA GAT TAC AA-3′ and 5′-GAG GGA GTT GGA AGG CTC TTC-3′ respectively. All results were normalized to succinate dehydrogenase complex subunit A (SDHA) using the 2^−ΔΔCt^ method [15]. Sequences for the SDHA forward and reverse primers were 5′-CAT GCT GCC GTG TTC CGT GTG GG-3′ and 5′-GGA CAG GGT GTG CTT CTT CCA GTG CTC C-3′ respectively.

### Western Blot

Effect of miR-1 overexpression on protein level was assessed 48 hours post transfection. Preadipocytes transfected with a non-targeting control siRNA or a miR-1 mimic (20 nM final concentration) were washed with PBS and harvested in a lysis buffer consisting of 10 mM Tris-HCl, 150 mM NaCl, 2 mM EDTA, 1% (v/v) Triton X-100, 10% (v/v) glycerol, 1 mM DTT and cOmplete Protease Inhibitor Cocktail from Roche. 30 µg protein lysates were separated by electrophoresis using 4–12% NuPAGE Novex Bis-Tris polyacrylamide gels (Life Technologies) and NuPAGE MES as running buffer. iBlot 7-Minute Blotting System form Life Technologies was used to achieve protein transfer onto nitrocellulose membranes. The membranes were blocked in 5% milk for one hour at room temperature. Incubations with primary antibodies to detect twinfilin-1 (1∶700 dilution) or α-tubulin (1∶5000 dilution) were followed by incubations with the appropriate secondary antibodies conjugated with HRP and by detection using ECL Western Blotting Detection Reagents and hyperfilms from Amersham Bioscience (Freiburg, Germany). Protein expression was quantified using ImageJ (U. S. National Institutes of Health, Bethesda, Maryland, USA) image processing software.

### Statistics

Comparisons between groups were computed with ANOVA using the GraphPad Prism software. Bonferroni's test was used for correcting multiple comparisons. P values <0.05 were considered statistically significant.

## Results and Discussion

The goal of this study was to produce a parallel comparison of the selected transfection agents in order to identify an efficient, non-viral transfection reagent capable of delivering active, non-coding RNAs (ncRNAs) into human preadipocytes and adipocytes. Several transfection reagents are currently available for the introduction of small, ncRNAs into cells but with adipocytes poor transfection rates are a frequent problem. A novel cationic lipid ScreenFect A has recently been shown to perform very well in production cell lines cultured in complex medium [16], a model, which like mature adipocytes, is described as difficult-to-transfect. In the same study, the authors found that a cationic polymer BPEI 1.2 k was efficient in terms of delivery but failed to maintain the functionality of the internalized RNA oligos. For the present study, we chose to compare ScreenFect A with the commonly used lipofection agent Lipofectamine 2000 and the polymer BPEI 1.2 k.

**Figure 1:**
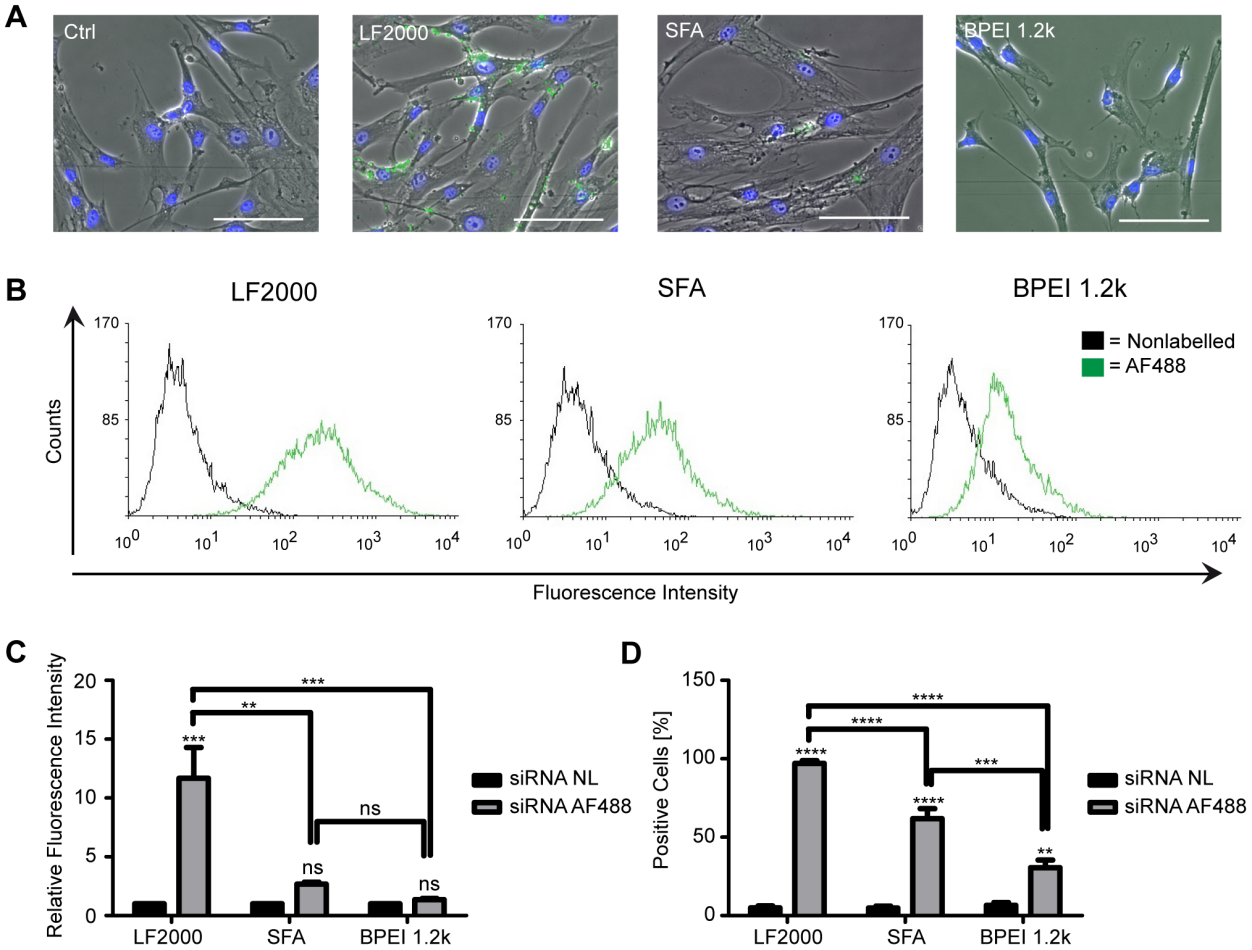
Delivery of siRNA into SGBS preadipocytes. Cells were transfected with a non-labelled and an Alexa Fluor 488 (green) labelled, nonspecific siRNA using Lipofectamine 2000 (LF2000), ScreenFect A (SFA) and BPEI 1.2 k as delivery agents. Uptake of the labelled siRNA was assayed 48 hours post-transfection with fluorescence microscopy and flow cytometry. Fluorescent overlay micrographs (A) of paraformaldehyde-fixed cells taken with a 20x objective. Nuclei were counterstained with DAPI (blue). Scale bars are 100 µm. Pictures from one representative experiment out of two performed are shown. Histogram overlays (B), relative fluorescence intensities (C) and transfection efficiencies (D) from the flow cytometric analysis of the siRNA AF488 transfected cells. Data are expressed as mean and SEM of three independent triplicate experiments. ***p<0.0005 LF2000 nonlabelled vs. labelled, **p<0.0021 LF2000 vs. SFA, ***p<0.0007 LF2000 vs. BPEI in (C), ****p<0.0001 LF2000 and SFA nonlabelled vs. labelled, **p<0.0015 BPEI nonlabelled vs. labelled, ****p<0.0001 LF2000 vs. SFA and LF2000 vs. BPEI and ***p<0.0002 SFA vs. BPEI in (D).

We first used an Alexa Fluor 488 labelled, nonspecific control siRNA (20 nM) to qualitatively assess transfection efficiency in SGBS preadipocytes via fluorescence microscopy (Figure 1A). While no signal was detectable in cells transfected with BPEI 1.2 k, a clear green fluorescent signal was observed inside the cells transfected either with Lipofectamine 2000 or ScreenFect A. DAPI co-staining indicated the siRNA to be localized in the cytoplasm preferably close to the nuclei. However, rather than being evenly distributed throughout the cytoplasm we always found the green signal to be present as concentrated dots. This may imply that the oligo is not efficiently released into the cytoplasm but is likely trapped in vesicles of the endosomal pathway. Whether this is due to inefficient release from the transfection agent:ncRNA complex or due to inadequate endosomal escape is unclear. Nevertheless, at least some free RNA must be present in the cytoplasm since we are able to demonstrate the downregulation of a specific gene in response to miRNA mimic overexpression as discussed later. Compared to a nonlabelled control, the flow cytometric analysis of preadipocytes transfected with the fluorescently-tagged siRNA revealed that the use of Lipofectamine 2000 resulted in highest fluorescence intensities in the analyzed cells (Figure 1B and C). This implies that Lipofectamine was able to internalize the highest amounts of oligo. Use of ScreenFect A or BPEI 1.2 k was associated with only modest fluorescence intensities which might also explain why the siRNA was not very prominent in the microscopic analysis with ScreenFect A and not detectable with BPEI 1.2 k. Quantification of the flow cytometry data indicated a close 100% transfection efficiency with Lipofectamine 2000 as presented in Figure 1D. With ScreenFect A the transfection efficiency was approximately 60% and 30% with BPEI 1.2 k.

**Figure 2:**
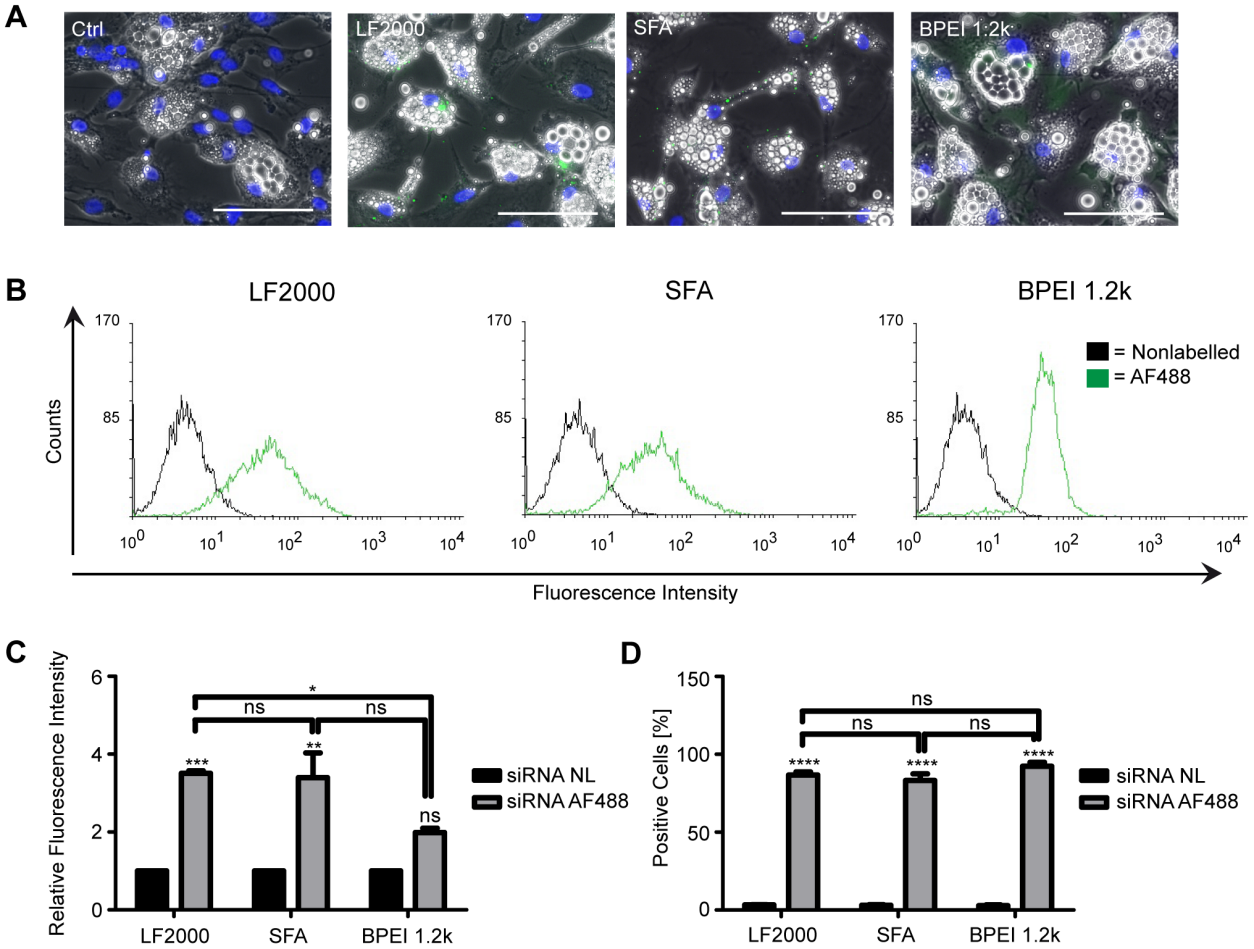
siRNA transfection of SGBS adipocytes. Cells were transfected with a non-labelled and an Alexa Fluor 488 (green) labelled, nonspecific siRNA using Lipofectamine 2000 (LF2000), ScreenFect A (SFA) and BPEI 1.2 k as delivery agents. Uptake of the labelled siRNA was assayed 48 hours post-transfection with fluorescence microscopy and flow cytometry. Fluorescent overlay micrographs (A) of paraformaldehyde-fixed cells taken with a 20x objective. Nuclei were counterstained with DAPI (blue). Scale bars are 100 µm. Pictures from one representative experiment out of two performed are shown. Histogram overlays (B), relative fluorescence intensities (C) and transfection efficiencies (D) from the flow cytometric analysis of the siRNA AF488 transfected cells. Data are expressed as mean and SEM of three independent triplicate experiments. ***p<0.0009 LF2000 nonlabelled vs. labelled, **p<0.0014 SFA nonlabelled vs. labelled and *p<0.0382 LF2000 vs. BPEI in (C) and ****p<0.0001 nonlabelled vs. labelled in (D).

Since adipocytes tend to be more resistant to transfection than preadipocytes, we were curious to see if the same transfection procedure could be successfully applied to adipocytes. Indeed, in terms of siRNA delivery the results with SGBS adipocytes were similar to the preadipocytes. The fluorescently-tagged control siRNA was present in the cytoplasm of adipocytes and seemed to be excluded from the lipid droplets (Figure 2 A) but as with the preadipocytes, concentrated spots were observed. Flow cytometric analysis of the transfected adipocytes (Figure 2 B, C and D) showed that the labelled siRNA was efficiently internalized by all three transfection agents. Transfection efficiencies for Lipofectamine 2000, ScreenFect A and rather surprisingly also for BPEI 1.2 k were around 90% but as with the preadipocytes, the difference between the three transfection agents could be seen from the fluorescence intensities. Cells transfected with Lipofectamine 2000 and ScreenFect A displayed higher fluorescence intensities than the ones transfected with BPEI 1.2 k indicating that the lipofection reagents were able to deliver higher amounts of the tagged oligo into adipocytes (LF2000 *vs.* BPEI p<0.04; SFA *vs.* BPEI p<0.07). There was no statistically significant difference between Lipofectamine 2000 and ScreenFect A.

**Figure 3:**
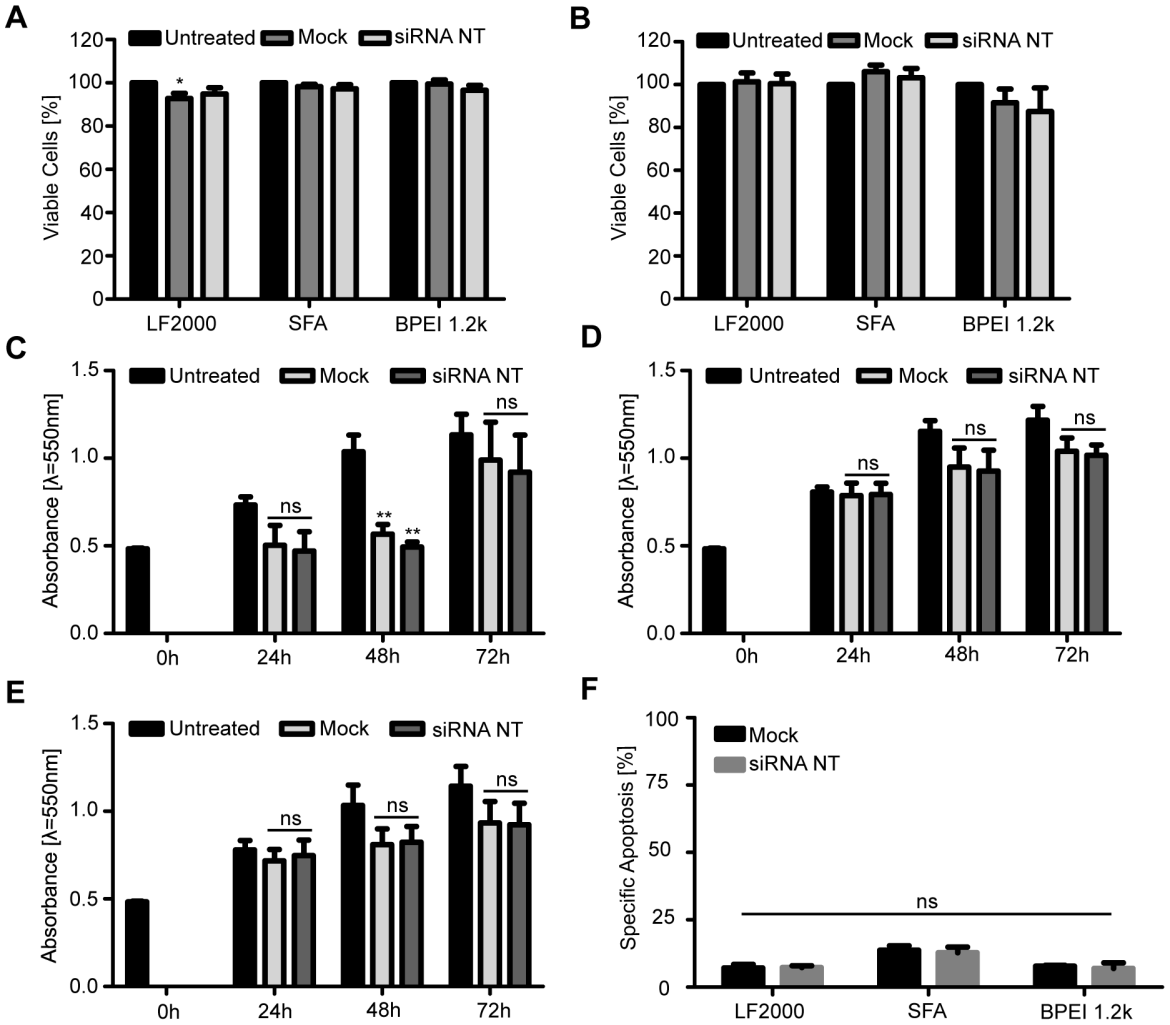
SGBS preadipocytes and adipocytes remain viable after siRNA transfection. Cells were left untreated, treated with transfection agent alone (mock) or transfected with a nontargeting (NT) control siRNA using Lipofectamine 2000 (LF2000), ScreenFect A (SFA) and BPEI 1.2 k as delivery agents. Cell viability was determined 48 hours post-transfection by detecting the production of calcein from calcein-AM via flow cytometry in (A) preadipocytes and (B) adipocytes. To probe the metabolic activity of the transfected cells, an MTT assay was performed 0, 24, 48 and 72 hours after preadipocyte transfection with (C) LF2000, (D) SFA and (E) BPEI 1.2 k as delivery agents. (F) Transfected preadipocytes were analyzed with flow cytometry 48 h post-transfection and the FSC/SSC profile of the cells was used to roughly investigate apoptosis induction. Specific apoptosis was calculated as explained in Methods. Three independent triplicate experiments were carried out. Results are reported as the mean and SEM. *p<0.0310 LF2000 untreated vs. mock in (A) and **p<0.0069 LF2000 untreated vs. mock and untreated vs. siRNA NT in (C).

Transfection procedures often cause cytotoxic effects. Therefore, we assessed the viability of the transfected SGBS preadipocytes and adipocytes using calcein-AM staining. Cells were left untreated, treated with a transfection agent alone (mock) or were transfected with a nonspecific control siRNA (20 nM). Proportion of live cells was determined 48 hours post-transfection by detecting the production of calcein from calcein-AM via flow cytometry (Figure 3 A and B). The vast majority (>85%) of both preadipocytes and adipocytes remained viable throughout the transfection procedure with all three delivery agents. This indicates that the lipid-based siRNA transfection with Lipofectamine 2000 and ScreenFect A and transfection with the cationic polymer BPEI 1.2 k are well tolerated by the SGBS cells. To further confirm this data, we performed MTT assays with the transfected SGBS preadipocytes. MTT measures mitochondrial activity as a surrogate marker of cell viability. Cells transfected with Lipofectamine 2000 showed a considerable decrease in mitochondrial activity 48 h after transfection, while ScreenFect A and BPEI 1.2 k had a modest effect (Figure 3 C, D and E). We therefore studied whether cell death is induced and analysed the FSC/SSC profile of these cells after transfection. We did not detect signs of early apoptosis in these analyses (Figure 3 F). We therefore conclude that the Lipofectamine 2000-induced decrease in the MTT assay was more likely due to a difference in cell proliferation and not due to cytotoxicity. This is supported by the fact that mitochondrial activity again increases after 72 h (Figure 3 C).

**Figure 4:**
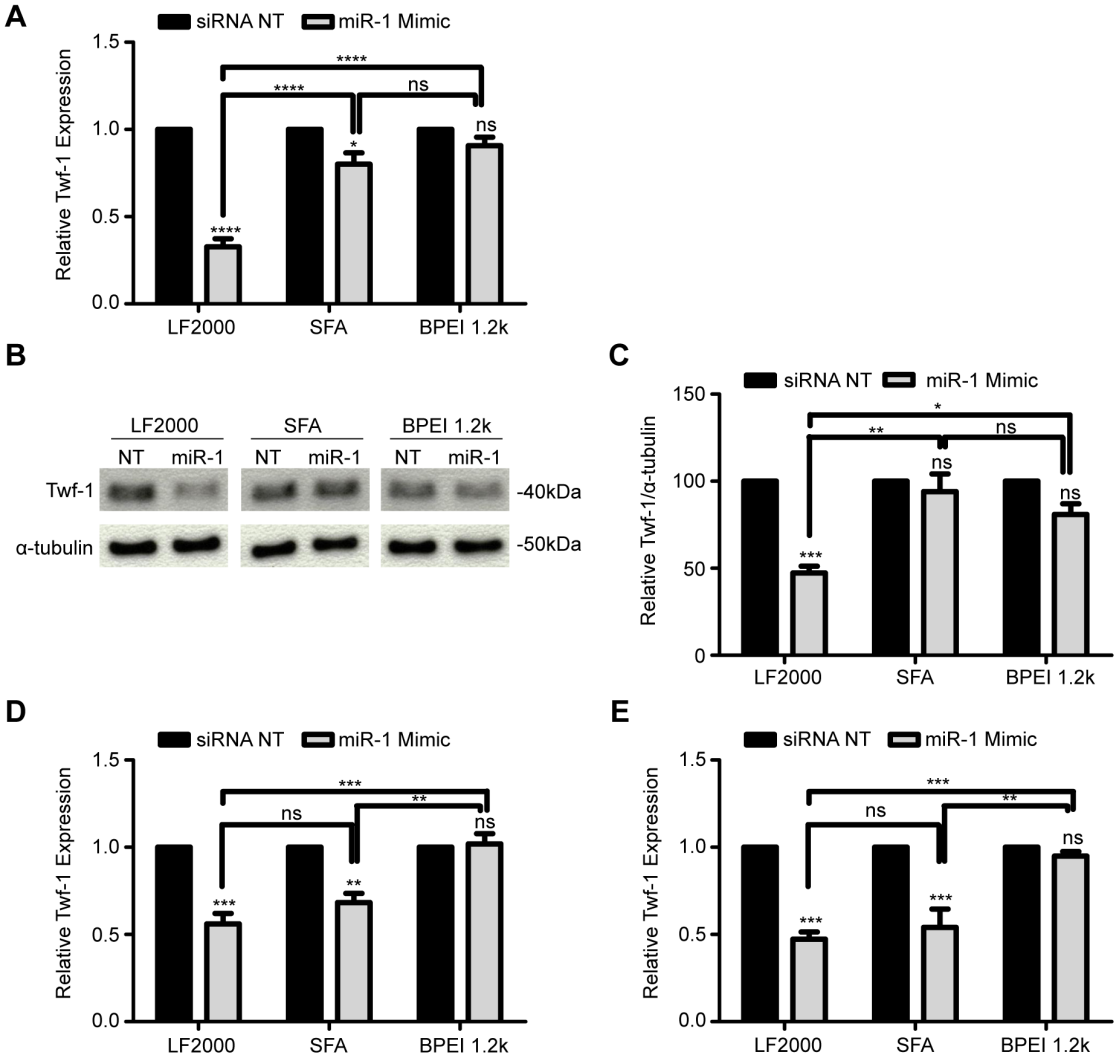
Lipid-based miRNA transfection is an effective method for targeted gene regulation in preadipocytes and adipocytes. SGBS preadipocytes were transfected with nontargeting (NT) siRNA and a miR-1 mimic with Lipofectamine 2000 (LF2000), ScreenFect A (SFA) or BPEI 1.2 k as delivery agents. (A) The levels of miR-1 target twinfilin-1 (twf-1) mRNA were determined with qPCR 48 hours post-transfection. (B) Protein samples were extracted 48 h after transfection and twf-1 protein levels assayed via western blotting and quantified using Image J software (C). 30 µg of protein was loaded in each lane. Equal loading was ensured by α-tubulin expression. SGBS adipocytes (D) and primary human adipocytes (E) were transfected with NT siRNA and miR-1 mimic with LF2000, SFA or BPEI 1.2 k. The levels of miR-1 target twinfilin-1 (twf-1) mRNA were determined with real-time qPCR 48 hours post-transfection. Experiments were carried out three times independently and the results are reported as mean and SEM. ****p<0.0001 and *p<0.0274 in (A), ***p<0.0007 LF2000 siRNA NT vs. miR-1, **p<0.0018 LF2000 vs. SFA and *p<0.0213 LF2000 vs. BPEI in (C), ***p<0.0002 LF2000 siRNA NT vs. miR-1, ***p<0.0006 SFA siRNA NT vs. miR-1, ***p<0.0005 LF2000 vs. BPEI and **p<0.0016 SFA vs. BPEI in (D), ***p<0.0004 and **p<0.0060 in (E).

A successful RNAi experiment is determined not only by efficient delivery, but also by the ability of the si/miRNA oligo to silence a specific target gene. Since our focus was on miRNAs, we searched for a suitable model mimic for the silencing experiments. miR-1 has been suggested to bind to the 3′-UTR of twinfilin-1 (twf-1) transcript and induce its mRNA degradation in human and mouse cells [17], [18]. We therefore performed a gene knockdown experiment where we tested the effect of increased cytoplasmic miR-1 abundance on the level of twf-1. After transfection with the miR-1 mimic (20 nM), the cells were incubated for 48 hours followed by total RNA extraction and reverse transcription reactions. qPCR was used to determine twf-1 mRNA levels relative to control samples of cells transfected with a non-targeting siRNA. In SGBS preadipocytes the most effective knockdown was achieved with Lipofectamine 2000 (Figure 4 A) which allowed for a 65% downregulation of the twf-1 gene on the mRNA level. Transfection of the miR-1 mimic using ScreenFect A reduced twf-1 mRNA expression by approximately 20%. The efficiency of knockdown using BPEI 1.2 k was negligible. These results were reflected also on protein level as Western blotting analysis revealed a clear twf-1 protein knockdown in response to the Lipofectamine 2000 delivered miR-1 overexpression (Figure 4 B and C).

To test the applicability of the twf-1 gene knockdown assay in mature adipocytes we used the three delivery agents to transfect SGBS adipocytes and also *ex vivo* differentiated primary human adipocytes with the miR-1 mimic. qPCR analysis revealed an efficient twf-1 downregulation in both cell types when using the two lipofection agents (Figure 4 D and E). With approximately 50% downregulation of the target gene, lipofection is a suitable method for loss-of-function studies in adipocytes using miRNA mimics. This result also confirmed that twf-1 is a target for miR-1 in adipocytes and provided us with an easily detectable positive control for further studies. Judging from the flow cytometry data, BPEI 1.2 k was able to deliver at least some RNA oligos into the preadipocytes and adipocytes but still no downregulation of a specific target gene was observed. The phenomenon is in line with the finding of Fischer *et al.* [16] and might be attributed to the very low numbers of RNA duplexes in the cells, which might not be sufficient to grant efficient gene downregulation.

**Figure 5:**
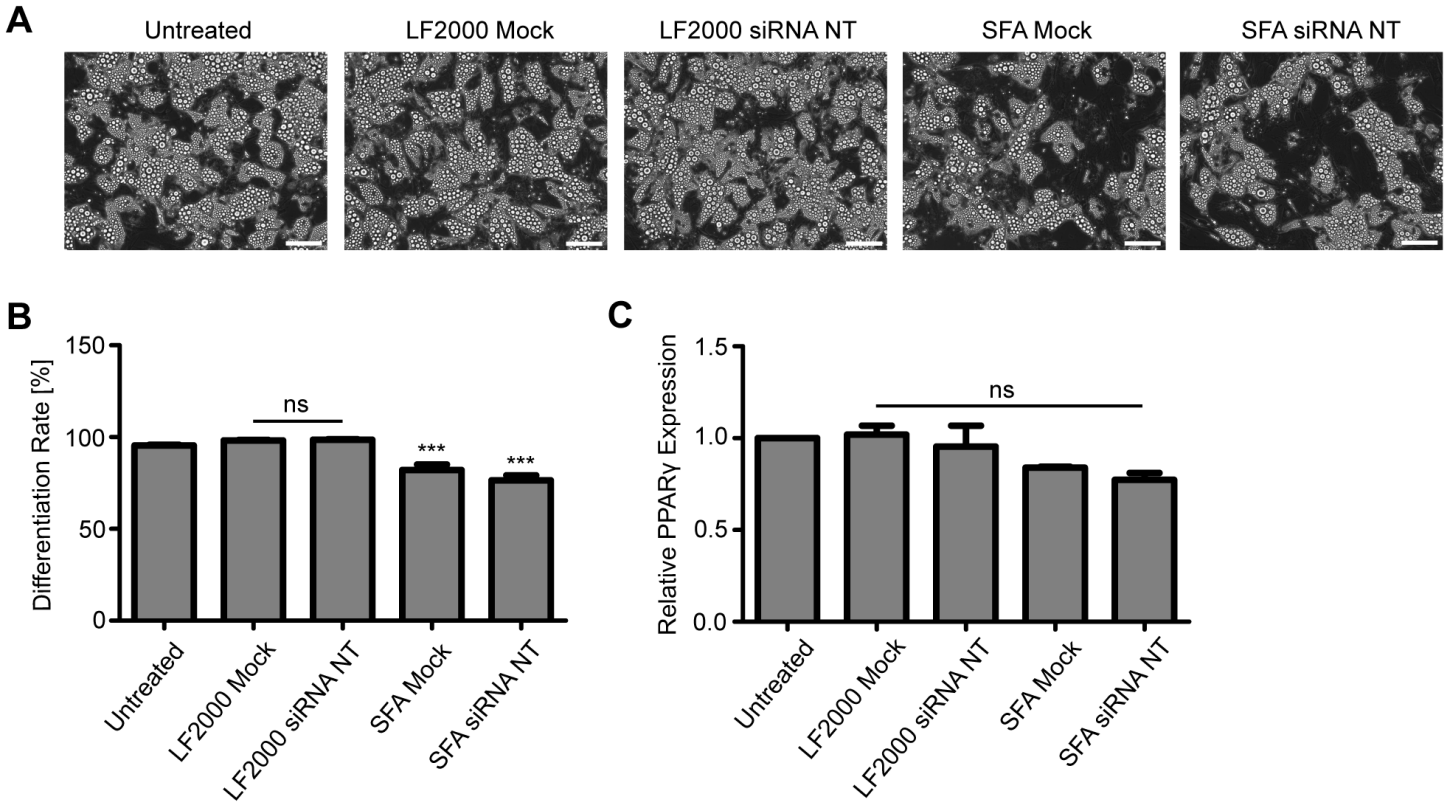
Transfected SGBS preadipocytes retain their ability for adipogenic differentiation. Untreated cells, cells treated with transfection agent alone (mock) and cells transfected with a nontargeting (NT) control siRNA were exposed to differentiation medium 48 h after transfection and analyzed on day 14 of adipogenic differentiation. Phase contrast micrographs (A) taken with a 20x objective. Scale bars are 100 µm. Pictures from one representative experiment out of two performed are shown. Differentiation rates (B) and the qPCR analysis of the expression level of an adipocyte marker gene PPARγ (C). The experiment was carried out three times independently and the results are expressed as mean and SEM. ***p<0.0001 untreated vs. SFA.

Finally, we were interested to see whether the transfected preadipocytes were still able to differentiate into adipocytes. BPEI was not studied further because it did not lead to a downregulation of target mRNA neither in SGBS preadipocytes nor in SGBS and primary adipocytes upon miR-1 mimic overexpression. The cells were left untreated, treated with the transfection agent alone or transfected with a non-targeting siRNA. Adipogenic differentiation was induced 48 h after transfection ( = day 0). The accumulation of lipids via light microscopy (Figure 5 A), differentiation rate (Figure 5 B) and upregulation of an adipocyte-specific marker PPARγ on the mRNA level (Figure 5 C) were monitored on day 14 of adipogenic differentiation. Compared to the control cells, Lipofectamine 2000 treated cells showed no disadvantages in differentiation while ScreenFect A had a mild inhibitory effect as judged by the impaired lipid accumulation, inferior differentiation rate and the tendency for lower PPARγ expression levels.

Taken together, our results show that lipid-based si/miRNA transfection of adherent SGBS preadipocytes and adipocytes is highly efficient as demonstrated by the cytoplasmic localization of the fluorescent-labelled control siRNA and the downregulation of a specific target twf-1 in response to miR-1 overexpression. The twf-1 knockdown was demonstrated also in *ex vivo* differentiated primary human adipocytes. Lipofection, therefore, is a feasible method for loss-of-function studies using miRNA mimics in human adipocytes. In terms of knockdown efficiency, Lipofectamine 2000 outperformed ScreenFect A in preadipocytes but in adipocytes the two compounds gave comparable results when used under the same conditions. Although Lipofectamine 2000 did not show overt cytotoxicity, ScreenFect A appears to be even less harmful to the cells and is also considerably more affordable. Further optimization might still improve its efficacy making it a notable alternative for the gold standard Lipofectamine 2000.
